# Age Friendly Care

**DOI:** 10.1093/geroni/igaf122.496

**Published:** 2025-12-31

## Abstract

This session addresses opportunities to improve Age Friendly health care using both the IHI 4 M’s and Geriatric Medicine’s 5 M’s and nursing’s gerontological competencies.

